# The Relationship Between Technology Use and Medication Access in Older Adults in Puerto Rico

**DOI:** 10.3390/ijerph22101534

**Published:** 2025-10-07

**Authors:** Joseph Badillo-Salcedo, Gabriela M. Vélez-Jiménez, Ethan G. Rosado-Martínez, Kyle Melin, Jonathan Hernández-Agosto

**Affiliations:** 1 School of Pharmacy, Medical Sciences Campus, University of Puerto Rico, San Juan, PR 00936, USA; joseph.badillo@upr.edu (J.B.-S.); gabriela.velez14@upr.edu (G.M.V.-J.); ethan.rosado@upr.edu (E.G.R.-M.); kyle.melin@upr.edu (K.M.); 2 National Alliance for Hispanic Health, District of Columbia, Washington, DC 20036, USA

**Keywords:** digitalization, health services accessibility, Hispanic or Latino, social determinants of health, digital literacy, pharmaceutical services, digital inequality, Geriatrics

## Abstract

The recent shift from in-person to digital pharmacy services is transforming how patients interact with their pharmacists but has the potential to disadvantage older adults (aged ≥ 65) who often face barriers when using technology. This study aimed to assess digital inequalities affecting medication access among older adults. A Spanish-language questionnaire was developed and psychometrically validated, revealing a two-factor latent structure comprising: (1) Technology Use, and (2) Medication Access. Item discrimination analysis confirmed that all significantly differentiated between those that used technology to facilitate their medication access and those that did not (*p* < 0.001). Participants with higher education reported greater income levels (*p* < 0.001), and income was in turn related to both internet access and digital skills. Age played a key role in perceptions of technology. Participants who considered technology helpful were younger ($\bar{x}$ = 72.9) than those who did not ($\bar{x}$ = 76.6; *p* = 0.001). There was no significant relationship between perceived technological usefulness and reporting not being able to acquire medications because of technological barriers (*p* = 0.788). This newly created and validated questionnaire identified gaps related to digital pharmacy services and may be a useful tool in future clinical, community, and investigative contexts.

## 1. Introduction

The evolution of digital services has transformed the landscape of healthcare delivery worldwide. Among these transformations, the digitalization of pharmacy services stands out for its potential to enhance medication access, improve adherence, and facilitate interactions with healthcare providers [1]. However, such digital transitions also introduce new challenges, particularly for populations that may lack digital literacy or socioeconomic support [2]. In Puerto Rico, a U.S. territory with persistent socioeconomic disparities and a rapidly aging population, these concerns are particularly salient [3]. Even more so when we consider that data related to medication access at a community pharmacy level in Puerto Rico is scarce and primarily based on anecdotal data from community pharmacies or extrapolated from U.S.-wide studies [4,5,6]. While digital inequalities have been observed in the provision of other types of healthcare services in the US and globally [7], they do not explore the unique context of how digitalization of pharmacy services may relate to medication access at a community pharmacy level.

Older adults (≥65 years) may face unique limitations due to factors such as limited digital literacy, educational barriers, and cognitive or physical limitations [8,9,10]. Such limitations may have been particularly pronounced following the COVID-19 pandemic, which led to limited social exposure and an acceleration in the transition of pharmacy services to “remote or at-home” options like telemedicine and eHealth [11,12]. Several key examples of these options include digital platforms for remote appointments, e-prescribing, and text message notifications for medication pick-up, refills, and medication adherence apps [13,14]. This rapid shift highlights the relevance of the proposed study, especially considering the limited knowledge available in the topic when it comes to Puerto Rico, which will assess how these changes may relate with medication access for older adults in Puerto Rico.

Identifying the factors associated with access to digital pharmacy services is relevant to understanding the needs of the target population and providing recommendations that safeguard patients’ access to medications [15]. A review of the existing literature yielded limited research on the topic, with no published, validated instruments tailored to the variables of interest. Several validated instruments were identified which contained related components to the research question at hand, including the E-Health Literacy Scale, e-Heals, and the Health Information Technology Usability Evaluation Scale [16,17], but none was tailored for this research specific objectives (in particular access to pharmacy services or medications) or validated in Spanish in a Puerto Rican population. Therefore, a Spanish-language instrument to measure the experiences of digitization of pharmacy services among older adults and identify related factors associated with access to these services was developed and validated. This result was a new instrument as an important output of the study, as it provides a new Spanish-language tool that researchers can use to better assess the association between the digitalization of pharmacy services and medication access in communities whose primary spoken language is Spanish.

In summary, this research aims to expand the limited knowledge on digital inequalities related to medication access among older adults by developing and utilizing an instrument that combines demographic data, use of technology, and access to medication parameters. Previous research has suggested that rapid digitalization may introduce barriers to access, particularly among those with lower educational attainment, socioeconomic status, and those living in rural communities. As such, this study is a further step toward examining how digital access and literacy comprise social determinants of health that shape medication access inequities in Puerto Rico. The three main objectives of this study were: (1) to evaluate the association of pharmacy service digitalization with medication access among older adults, (2) to identify socio-demographic and structural factors that influence digital access to these services by older adults, and (3) to propose recommendations for mitigating digital exclusion in pharmacy services in this population.

## 2. Materials and Methods

### 2.1. Study Overview

In this observational, cross-sectional study, we created, pilot-tested, and revised a questionnaire across two major phases. During Phase 1, the questionnaire was developed and pilot tested as a research instrument to assess older adults’ access to medication in the context of digital pharmacy services. Meanwhile in Phase 2, the questionnaire was validated and applied in a real-world context to evaluate how digitalization relates to medication access across Puerto Rico’s elderly population.

#### Sample Size Justification and Recruitment Strategy

Researchers conducted an *a priori* power analysis to determine the adequacy of the sample size for chi-square tests of independence. Python 3.12.2 and the library Statsmodels 0.14.2 were used to compute the required sample size, assuming a medium effect size (Cohen’s w = 0.20), an alpha level of 0.05 (two-sided) and power (1 − β) = 0.80. The required sample size calculated using the conventional normal-approximation formula was approximately 197 participants. To account for incomplete responses and increase robustness, recruitment targeted a larger sample and resulted in a final sample of 307 older adults, thus exceeding the minimum required by the power analysis. This sample size ensured adequate power to detect small-to-moderate associations between socio-demographic characteristics, technology use, and medication access through pharmacy services.

Based on recent estimates, Puerto Rico has approximately 789,000 adults aged 65 years or older, representing nearly one-quarter (24.6%) of the total population [18]. While not intended to be statistically representative of all older adults in Puerto Rico, this sample provides sufficient variability to explore key patterns in technological use and its relationship with medication access within this age group.

Participants were recruited through a convenience sampling strategy across diverse settings, including community pharmacies, churches, and other public spaces across Puerto Rico. Collaborating pharmacies and community organizations facilitated access by allowing the research team to approach older adults directly on-site. This approach was chosen to maximize geographic and demographic diversity while ensuring accessibility for older adults who might not otherwise engage with online recruitment methods.

### 2.2. Phase 1: Instrument Development and Validation

#### 2.2.1. Item Creation

The initial version of the Spanish-language questionnaire included 35 items (11 sociodemographic and 24 construct-measurement); some of them adapted from the E-Health Literacy Scale, e-Heals, and the Health Information Technology Usability Evaluation Scale [16,17] to consider the older adults’ population of Puerto Rico, as well as several created by the study team. When creating and selecting these items, sociodemographic factors that may influence technology usage and skill like income, age, and education level were considered as to include questions that allowed us to analyze an encompassing sociodemographic profile of the participants. Similarly, it was considered that not only questions for general technology use were to be developed, but also technology use specifically related to pharmacy services. This distinction was considered because, while individuals may have internet access and an electronic device to access apps and the web, it does not mean they may be skilled using them both in general terms and to access pharmacy services. For example, an individual may have such a device and use it for social media and messaging but may have problems using a website to schedule a vaccine appointment. After these elements were considered, the resulting items were divided across two domains (Technology Use and Medication Access) and a Sociodemographic Data section, with the purpose of understanding the relationships between sociodemographic factors, technology use, and their association with access to medications.

#### 2.2.2. Item Revision: Content Validity

Following item creation, the questionnaire, which was created specifically for older adults’ population, was submitted to review by five experts in pharmacy, education, gerontology, and social work, to ensure its content validity. The experts evaluated the questionnaire based on the relevance, clarity, and representativeness of each of its items. The content validity procedure led to the retention of 32 of the original 35 items in the questionnaire, with minor revisions being needed to improve clarity and adapt wording for better cultural relevance.

#### 2.2.3. Item Revision: Cognitive Interviews

Once the questionnaire was modified based on the input from the panel of experts, it was used to conduct thirty-minute interviews with ten participants who met the study’s inclusion criteria. These thirty-minute interviews were carried out by the investigators and used paper-printed copies of the questionnaire. During this process, the participants were prompted to verbalize their thought process while answering each item, which allowed the research team to assess comprehension, identify ambiguous phrasing, and ensure cultural appropriateness. The insight provided by the interviews led to linguistic adjustments and clarification in several items, particularly those related to digital platforms and service access pathways.

#### 2.2.4. Pilot Testing

During the pilot testing phase, the revised version of the questionnaire was administered to a sample of fifty-one participants who met the inclusion criteria: 65 or more years of age and have taken or been prescribed at least one medication in the past 6 months. Unlike the cognitive testing phase, this administration followed the same standardized format intended for the main data collection phase—participants completed the questionnaire independently with minimal intervention from investigators. This approach was designed to simulate real research conditions and assess the instrument’s functionality in its final delivery format.

#### 2.2.5. Item Reduction and Factor Extraction

**Figure 1:**
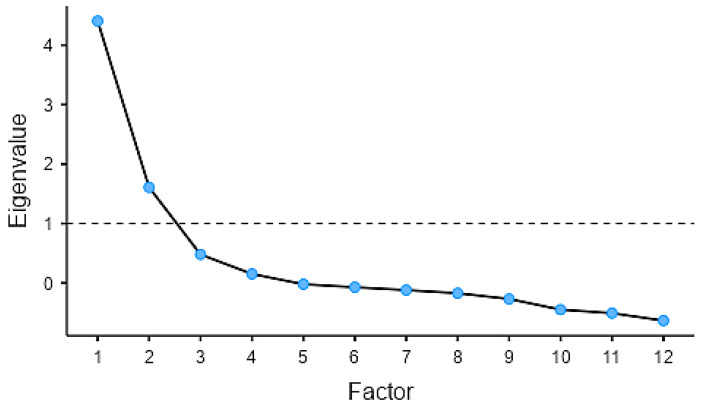
Scree plot. The blue dots in the scree plot represent the eigenvalues associated with each factor in the factor analysis. Each eigenvalue indicates the proportion of variance explained by that factor. The dashed line indicates the model fit or estimated trend based on the analysis.

The data collected during pilot-testing phase supported the Exploratory Factor Analysis (EFA) and provided preliminary evidence of the instrument’s structural validity and clarity, which was conducted using Jamovi 2.6.17. An EFA on the twenty-four construct-related items using principal axis factoring with oblimin rotation was performed. After item reduction, Bartlett’s test indicated factorability, χ^2^ (66) = 388, *p* < 0.001. Twelve items were retained for a two-factor structure, based on eigenvalues > 1, scree plot (see Figure 1), and variance explained (31.4% and 22.5%, cumulative 54%).

The two-factor confirmatory factor analysis model showed an acceptable fit to the data: χ^2^ (43) = 79.3, *p* < 0.001, RMSEA = 0.127 (90% CI [0.084, 0.174]), TLI = 0.820, and BIC = –89.8. Factor 1 (*Technology Use*) comprised six items, with standardized loadings ranging from 0.60 to 0.96, indicating strong associations with the underlying construct. Factor 2 (*Medication Access*) also included six items, with loadings ranging from 0.35 to 0.98 (see Table 1). While most items demonstrated satisfactory loadings, some in the *Medication Access* subscale showed relatively weaker associations.

These results support a two-factor structure, though fit indices suggested further refinement in confirmatory analysis. Therefore, factor extraction procedures yielded a refined 12-item version of the questionnaire structured around two distinct and interpretable constructs: *Technology Use* (6 items) and *Medication Access* (6 items) via Kaiser-Meyer-Olkin (0.770) and the Tucker–Lewis Index to confirm structural adequacy [19,20]. The items which presented a load of <0.3, uniqueness > 0.8, and null variance were eliminated.

### 2.3. Phase 2: Instrument Implementation and Confirmatory Factor Analysis

Following completion of Phase 1, the refined questionnaire was administered to 307 participants who met the study’s inclusion criteria. The 307 surveys were administered over a 2-month time frame and included the support of local community pharmacies and churches that made their facilities available for the participant recruitment process.

Once the interventions were completed, a Confirmatory Factor Analysis (CFA) was carried out with a bidimensional model and correlated factors using Python 3.12.2 and the libraries FactorAnalyzer 0.5.1 and Semopy 2.3.11, within the Positron IDE 2025.09.0-139. The 4th item of the second factor (S2-Q4): “¿Alguna vez ha dejado de pedir o recoger un medicamento porque no pudo utilizar las herramientas tecnológicas que su farmacia requería?” [English Translation: “Have you ever stopped requesting or not picked up your medications because you couldn’t use the technology platforms required by your pharmacy?”], was excluded from the CFA because of a lack of variability in responses. The exclusion of item S2-Q4 improved the internal consistency of the *Medication Access* subscale, increasing McDonald’s ω from 0.55 to 0.61. However, this item responses were still kept for descriptive purposes. A second analysis was then carried out with McDonald’s omega factor (ω), preferred for ordinal data [21,22], to evaluate once more the internal validity of the refined questionnaire with the bigger sample of the implementation phase.

The two-factor confirmatory factor analysis model showed an acceptable fit to the data: χ^2^ (43) = 135.09, χ^2^/df = 3.14, CFI = 0.938, TLI = 0.921, and RMSEA = 0.084 (90% CI [0.063, 0.106]). Factor 1 (*Technology Use*) comprised six items (S1-Q1 to S1-Q6), with standardized loadings ranging from 0.579 to 0.945, indicating generally strong associations with the underlying construct. Factor 2 (*Medication Access*) included five items (S2-Q1 to S2-Q6, excluding S2-Q4), with loadings ranging from 0.172 to 0.730 (see Table 2). While most items demonstrated acceptable loadings, S2-Q6 showed a particularly weak association, suggesting that this subscale may benefit from item revision or refinement.

These CFA results align with the internal consistency findings using McDonald’s ω, which indicated satisfactory reliability for the *Technology Use* subscale (ω ≈ 0.907) and lower reliability for the *Medication Access* subscale (ω ≈ 0.612). The reduced consistency of the *Medication Access* subscale likely reflects both its heterogeneous item content and the limited number of items, highlighting areas for potential scale improvement.

Finally, a *t*-test on item discrimination analysis was carried out considering the extreme groups as those in the upper 27% and lower 27% to evaluate how well the items discriminate between high and low performers in both factors. The *Technology Use* scale, was developed to discriminate between those with noticeable ability and access to the technological tool versus those with limitations. Meanwhile, the *Medication Access* scale, was developed to discriminate between those that use technology to facilitate their access to their pharmacotherapy and those who did not. All items significantly differentiated (*p* < 0.000) between participants who reported using technology to facilitate medication access and those who did not, supporting the internal validity of the instrument.

Moreover, the McDonald’s omega (ω) was calculated to assess the internal consistency of the revised questionnaire. The overall scale had a ω = 0.895, exceeding the commonly accepted threshold of 0.70 for adequate reliability [21,22], thereby supporting the internal consistency of the final instrument.

Table 3 shows a summary of the internal consistency measures of the questionnaire scales developed for this study. The combined final version shows consistent measurement properties, supporting its validity for assessing the constructs of technology use and medication access (see Supplementary Materials for final questionnaire version, tables and figures). 

### 2.4. Recruitment and Protection of Human Subjects

The population of interest for this study were older adults living in Puerto Rico. Specific inclusion criteria included the following parameters: 65 or more years of age and have taken or been prescribed at least one medication in the past 6 months. The subjects for this study were selected among public spaces and collaborating centers like pharmacies and churches via convenience sampling.

This study went through expedited review by the University of Puerto Rico Medical Sciences Campus Institutional Review Board (Review number 2411312691). A waiver for written, informed consent was obtained from IRB and all participants received a written consent cover letter included at the beginning of the paper-printed copies of the questionnaire. This cover letter informed participants about what the study entailed, that their participation was voluntary and anonymous and their responses were confidential, and that by completing the survey, they were providing their consent to participate in the study. Once the subject completed the questionnaire, the data was digitized via a Google Form in a database that is stored in the investigators’ personal accounts and devices which are password, two-factor authentication, and biometrically protected. After 5 years, the data will be permanently deleted from the investigators’ devices.

### 2.5. Data Analysis

After completion of data collection process, an initial descriptive analysis was performed using both subscales results and the demographic data domain of the questionnaire. This was followed by an evaluation of sociodemographic differences by the Shapiro–Wilk test for normality. Similarly, a non-parametric pairwise Dwass–Steel–Critchlow–Fligner test was used to consider if there was a significant variation between male and female participants.

After this initial evaluation of sociodemographic differences, the *Technology Use* and *Medication* Access subscales were evaluated using a chi-square test of independence, in which *p* < 0.05 was considered as a representation of statistical significance. Similarly, the influence of age and geographic region on perceived technological access was also evaluated. The sociodemographic factor of age was initially evaluated using an independent sample *t*-test to evaluate if there were differences in perceived benefits of technology use, followed by a logistic regression model to further examine the potential predictive association of age and perceptions of benefits of technology on medication access. The potential correlation between geographic regions and perceived access to medications via technology was evaluated via chi-square test of independence, and the robustness of the sample being ensured via a Monte Carlo simulation with 10,000 replicates. Finally, a standardized heatmap was also carried out to better represent this data by region.

### 2.6. Limitations of the Study

The most notable limitation of the study was that the samples for both phases were selected using convenience sampling, which limited the generalizability of the results. This reliance on convenience sampling significantly affects the ability to generalize the findings to the broader population of Puerto Rico as the people recruited at community pharmacies may differ from more digitally marginalized older adults, like those critically ill, homebound, or socially isolated.

## 3. Results

This study involved 307 subjects from different regions of Puerto Rico. The characteristics of the 307 participants in the study can be seen in Table 4. Participants’ ages ranged from 65 to 98, with a mean age of 73 years. Reported gender was relatively balanced with 60% of participants identifying as female. The highest educational level was somewhat broadly distributed with 24% reporting a bachelor’s degree, 21% reporting a graduate degree, and 55% reporting some college courses, a high school education, or less. More than half of the participants reported to be retired (64%) and 71% of the participants reported living accompanied by their family.

Section two of the questionnaire explored participant’s general access and interaction with digital technologies. Table 5 presents the distribution of responses regarding participants’ general technology use. Most respondents reported having internet access at home (78.2%) and using mobile phones as their primary device for accessing the internet (77.1%). However, digital proficiency remained relatively low, with participants, on average, reporting “little ability” to use technology. Internet use was also limited in frequency, with most reporting use “more than once a week” but not daily. When asked about the frequency of general technology use, the average response was “once a week,” and only 69.6% reported using applications on their mobile devices. These results suggest that while access to digital tools is relatively widespread among older adults in Puerto Rico, consistent and confident usage remains low, which may have implications for their engagement with technology-based pharmacy services.

A summary of participants’ responses related to their interaction with pharmacy services through digital platforms are presented in Table 6. While 78.1% of participants reported believing that technology facilitates access to their medication therapy, only 58.5% indicated that they use devices to receive pharmacy-related services, and just 26.6% reported using electronic platforms to access such services directly. A significant proportion (66.9%) stated that they do not use these platforms, and an additional 6.6% reported that their pharmacy does not offer them. Notably, 9.8% of respondents indicated having failed to request or pick up medication due to technological barriers. Regarding preferred methods for obtaining pharmacy services, most participants favored doctor-initiated prescriptions (33.6%) and in-person visits (30.6%), with fewer opting for phone calls (25.4%) or text messages (5.2%). These findings highlight a discrepancy between positive perceptions of digital tools and their actual use, suggesting potential barriers related to access, awareness, or usability.

### 3.1. Technology Use and Access

We compared several indicators of technology use and demographic characteristics (Table 7). There was a strong and significant association between participants’ self-reported technology skill level and whether they have internet access at home (*p* < 0.001). Participants with home internet access were significantly more likely to have higher digital skills. Meanwhile, there was no significant difference between genders in terms of how often they use technology for pharmacy-related tasks (*p* = 0.241) or how frequently participants use the internet (*p* = 0.529).

On the other hand, a chi-square test of independence showed that there was no statistically significant relationship between having missed medications due to digital barriers and believing that technology improves access to medications (χ^2^ = 0.0723, *p* = 0.788; Table 8).

To further investigate whether educational attainment influences perceptions about the benefits of technology in pharmacy access, a contingency table analysis was performed. The chi-square test for association approached statistical significance (χ^2^ = 13.7, df = 7, *p* = 0.056), and Fisher’s exact test, conducted with Monte Carlo simulation for increased robustness, yielded a statistically significant result (*p* = 0.047). These findings indicate that participants with higher educational levels were significantly more likely to perceive technology as helpful for accessing their medication therapy. This reinforces the notion that educational background may shape individuals’ comfort, confidence, and perceived utility of digital pharmacy services.

### 3.2. Influence of Age and Geographic Region on Perceived Access

To better understand the relationship between sociodemographic factors and perceptions of technological access to medications, additional statistical analyses were performed using responses to Item S2-Q5: “¿Piensa que el uso de la tecnología para obtener servicios farmacéuticos le facilita el acceso a su terapia de medicamentos?” [English Translation: “Do you think that using technology to obtain pharmacy services facilitates access to your medication therapy?”]. First, an independent sample *t*-test was conducted to assess whether age influenced perceived benefit. The analysis revealed a statistically significant difference in mean age between respondents who answered “Yes” and those who answered “No”; participants who perceived a benefit were younger on average (M = 72.9 years) than those who did not ($\bar{x}$ = 76.6 years, *p* = 0.001). A logistic regression model was subsequently developed to further explore the predictive association of age on perceived benefit. The model was statistically significant (*p* < 0.001, pseudo-R^2^ = 0.040), with a negative regression coefficient for age (β = −0.066, *p* < 0.001), indicating that each additional year of age decreased the odds of perceiving technology as a facilitator of medication access by approximately 6.6%.

**Figure 2:**
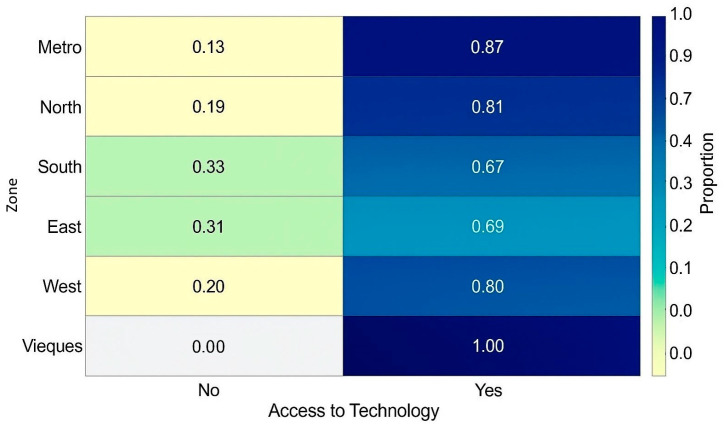
Standardized proportion heat map of perceived ease of access by geographic zone.

Additionally, a chi-square test of independence was performed to evaluate the association between geographic region and perceived access to medications via technology. This test yielded a statistically significant result (χ^2^ (6, *n* = 302) = 15.403, *p* = 0.0173), suggesting regional differences in perceptions. To ensure robustness, a Monte Carlo simulation with 10,000 replicates was conducted, which confirmed the statistical significance (adjusted *p* = 0.018). A standardized proportion heat map (Figure 2) further revealed that the West region had a higher-than-expected number of negative responses, while participants from Metro and Vieques were more likely to report that technology improved access. These results highlight the intersection of age and geography as key factors shaping digital health engagement among older adults in Puerto Rico.

## 4. Discussion

This study explored the intersection of pharmacy digitalization and sociodemographic inequalities among older adults in Puerto Rico. The results support the initial hypothesis: while digital tools are widely perceived as helpful, they do not equitably improve medication access for all older adults, particularly those facing economic or structural disadvantages. These findings echo global concerns that digital health transformations, without inclusive policy and infrastructure, may reinforce rather than reduce healthcare disparities [23,24]. Situated within a Latino community, this study emphasizes how digital inclusion is not only a technological issue but also a fundamental social determinant of health with implications for equity in medication access. The development of a Spanish-language questionnaire validated in this study is an additional significant output, as it provides a tool that can be used by other researchers to reach older adults in Spanish-speaking communities as well as those who immigrated to non-Spanish speaking countries where language may persist a barrier.

These results are some of the first to describe medication access at a community pharmacy level in Puerto Rico as well as the impact of digitalization on medication access. Evaluating how digital pharmacy services affect access to medications highlighted a revealing disconnect. Although most participants viewed digital tools positively, around 10% reported missing medications due to technological barriers. This discrepancy may stem from factors such as low digital literacy, cognitive burden, interface complexity, or limited support systems, an issue also observed by Frydman et al., who reported that older adults with significant health needs were less likely to access telemedicine despite the potential benefits [25].

On the other hand, addressing sociodemographic and structural barriers, the study found that income and education were significantly associated. Participants with higher educational attainment reported greater income levels, and income was in turn related to both internet access and digital skill. To directly assess whether education influenced perceptions of access, a contingency table analysis was conducted between participants’ education level (D-Q7) and their response to whether technology facilitates access to medications (S2-Q5). The results suggest that individuals with higher educational levels were significantly more likely to perceive technology as a facilitator of medication access. For instance, while only 65% of participants with primary education responded “Yes,” this rose to 83% among those with a bachelor’s degree and 96% among those with postgraduate education. These findings highlight a potential digital divide, where educational background influences perceived benefit and likely comfort in managing health needs through digital pharmacy services. This aligns with the findings by Choi and DiNitto, who emphasized the role of income and race/ethnicity in limiting eHealth use among older, homebound individuals [26]. In contrast, Lopez de Coca et al. observed that education and urban residence were stronger predictors of pharmacy-based digital engagement than gender, a finding mirrored in this study, which showed no significant gender differences in internet or pharmacy technology use despite income differences [23].

Further analyses revealed that age plays a key role in shaping these perceptions. Participants who considered technology helpful were significantly younger (mean age 72.9) than those who did not (mean age 76.6). Each additional year of age reduced the odds of perceiving digital tools as beneficial by approximately 6.6%. This age gradient aligns with existing literature on cognitive and technological barriers among older seniors, underscoring the importance of tailoring digital health tools to varying levels of familiarity and comfort with technology [27]. Furthermore, geographic disparities were also observed. A chi-square analysis confirmed a statistically significant association between region of residence and perceived access via technology, with participants in the West zone reporting lower-than-expected positive responses, while those in the Metro region and Vieques were more likely to report that technology helped them access medications. These patterns may reflect variations in infrastructure, availability of pharmacy services, or community-based digital support. The regional component adds another layer of complexity to the digital divide, highlighting the need for localized interventions that consider both access and contextual usability. Overall, these findings suggest that interventions should prioritize those with lower income and education. Accessibility, ease of navigation, and user support must be built into pharmacy platforms from the design phase. These implications are consistent with the findings of Kebede et al., who conducted qualitative interviews with older adults and caregivers and emphasized that both individual ability and broader infrastructural conditions like reliable internet determine digital engagement [28]. They also highlighted the role of caregivers, who frequently act as digital mediators, a strategy that this study suggests could be leveraged more systematically in Puerto Rico. Finally, Wu et al. demonstrated that consistent access to internet tools meaningfully reduced health disparities among older Chinese adults, further emphasizing the potential public health benefit of investing in inclusive digital infrastructure in underserved areas like rural Puerto Rico [24]. This supports the need for local policy that recognizes access to digital tools not merely as a convenience, but as a determinant of health.

We note several limitations that should be considered when considering the results of this study. This study employed a convenience sampling methodology to select participants from community venues and pharmacies. Hence, the interpretation of these results may have led to the underrepresentation of older adults who are most digitally marginalized (e.g., critically ill, homebound, or socially isolated), thereby limiting the generalizability of our findings to the entire older adult population of Puerto Rico. Future studies employing probabilistic sampling strategies could enhance external validity. Additionally, the *Medication Access* subscale demonstrated relatively low internal consistency (McDonald’s ω ≈ 0.55), which warrants consideration. This reduced reliability likely stems from two key factors: the limited number of items included in the subscale and the heterogeneous nature of the construct itself, which encompasses diverse dimensions of access such as affordability, availability, and logistical barriers. These aspects may not be uniformly captured by the current item set, leading to weaker inter-item correlations. Future iterations of the scale could benefit from expanding the number of items and refining their content to better represent the multidimensionality of medication access. Additionally, item S2_Q6, which showed particularly weak factor loading, may require revision or replacement to enhance the overall coherence of the subscale.

## 5. Conclusions

The digitalization of pharmacy services presents significant opportunities to enhance healthcare delivery; however, this study demonstrates that older adults in Puerto Rico do not experience these benefits equally. Despite general perceptions of usefulness, structural barriers such as limited income, lower educational attainment, and restricted internet access hinder equitable engagement with digital pharmacy platforms. These findings underscore the need for targeted digital literacy initiatives, inclusive system design, and public policies aimed at closing access gaps. To ensure that digital health tools fulfill their potential as equalizers rather than amplifiers of disparity, future studies should employ longitudinal and multivariate designs that more accurately capture causal relationships between structural determinants and digital engagement. Additionally, incorporating qualitative methodologies could offer deeper insight into how older adults experience digital pharmacy tools in their daily lives, paving the way for more user-centered, culturally responsive solutions. Taken together, these findings highlight how digital access and literacy operate as critical social determinants of health in Latino communities, aligning with broader public health efforts to reduce disparities. Achieving digital equity in pharmacy services will require coordinated efforts across technological, educational, and policy domains.

## Figures and Tables

**Table 1:** Factor loadings after EFA.

	Factor Loadings	
Retained Items	Technology Use	Medication Access
S1-Q2	0.598	
S1-Q3	0.848	
S1-Q4	0.957	
S1-Q5	0.709	
S1-Q6	0.784	
S1-Q8	0.751	
S2-Q1		0.977
S2-Q2		0.531
S2-Q3		0.350
S2-Q9		0.857
S2-Q10		0.513
S2-Q11		0.417

**Table 2:** Factor loadings after CFA.

Items	Factor Loadings	
	Technology Use	Medication Access
S1-Q1	0.7130783	
S1-Q2	0.7909485	
S1-Q3	0.9445337	
S1-Q4	0.5785176	
S1-Q5	0.8345147	
S1-Q6	0.8272698	
S2-Q1		0.730028
S2-Q2		0.447732
S2-Q3		0.565492
S2-Q5		0.491483
S2-Q6		0.1724121

**Table 3:** Subscale internal consistency (ω).

Subscale	No. of Items	ω
Technology Use	6	0.907
Medication Access *	5	0.612
Overall Scale	11	0.895

* The final questionnaire includes a sixth question in this scale. The question was not included in the CFA due to low variability in respondents but kept in the final instrument as it served a descriptive purpose.

**Table 4:** Participant characteristics (*n* = 307).

Variable	*n* (%)
Age (years)	73.7 (mean) ± 7.5 (std. dev.); 73 (median)
Gender *	
Woman	184 (60%)
Man	123 (40%)
Highest educational level	
Bachelor’s	74 (24%)
Graduate	64 (21%)
Some College, High School, or Less	169 (55%)
Employment status	
Retired	196 (63.8%)
Disabled	54 (17.6%)
Employed	33 (10.8%)
Homemaker	17 (5.5%)
Monthly income	$2078 (mean) ± $2672 (std. dev.); $1400 (median)
Type of income	
Individual	242 (78.8%)
Couple	49 (16%)
Other	16 (5.2%)
Living situation	
Alone	89 (29%)
Accompanied	218 (71%)
Assistance managing medications	
With help	134 (43.7%)
Without help	173 (56.3%)
Frequency of visits to providers	2.3 visits/month (mean)

* “Non-Binary” and “Other” were available as options; none of the respondents selected them.

**Table 5:** Access and use of technology (*n* = 307).

Variable	*n* (%)
Ability to use technology *	Median: I have little ability
Access to internet	
Yes	240 (78.2%)
No	67 (21.8%)
Frequency of internet use **	Median: More than once a week
Device used to access the Internet	
Phone	236 (77.1%)
Computer	52 (17%)
Tablet	49 (16%)
None	65 (21.2%)
Frequency of technology use **	Median: Once a week
Used applications on the mobile device	
Yes	214 (69.6%)
No	93 (30.4%)

* Answer choices: I have a lot of ability, I have ability, I have little ability, I have no ability. ** Answer choices: Never, Once a month, Once a week, More than once a week, Once a day, More than once a day.

**Table 6:** Access to medications (*n* = 307).

Variable	*n* (%)
Uses electronic devices to receive pharmacy services	
Yes	180 (58.5%)
No	127 (41.5%)
Frequency of automatic notifications from pharmacy	
Once a month	156 (50.8%)
Never	116 (37.7%)
Other	35 (11.5%)
Uses electronic platforms to access pharmacy services	
Yes	82 (26.6%)
No	205 (66.9%)
Pharmacy doesn’t use it	20 (6.6%)
Failed to order or pick up medication because of technology	
Yes	30 (9.8%)
No	242 (78.8%)
Don’t remember	16 (5.2%)
Thinks technology helps access pharmacy services and medication therapy	
Yes	240 (78.1%)
No	67 (21.9%)
Favorite option for obtaining pharmacy services	
Electronic platforms	0 (0%)
Phone call	78 (25.4%)
Text message	16 (5.2%)
Physician sent Rx	103 (33.6%)
Visiting the pharmacy	94 (30.6%)
Other	16 (5.2%)

**Table 7:** Technology Use indicators by participant group.

Variable	χ^2^ (df)	*p*-Value
Internet Access vs. Technology Skill Level	99.7 (3)	<0.001
Technology Use for Pharmacy vs. Gender	1.37 (1)	0.241
Internet Use Frequency vs. Gender	4.14 (5)	0.529

**Table 8:** Barrier perception and technology helpfulness.

Missed Medications	Believes Tech Helps	Total
Yes (*n* = 30)	24 (80%)	30
No (*n* = 271)	211 (77.9%)	271
Total	235 (77.9%)	301

## Data Availability

The dataset generated for this study is not publicly available due to confidentiality provisions provided to participants upon enrollment in the study. Data are available from the authors upon reasonable request with approval by the University of Puerto Rico—Medical Sciences Campus Institutional Review Board.

## Supplementary Materials

The following supporting information can be downloaded at: https://www.mdpi.com/article/10.3390/ijerph22101534/s1, Final Questionnaire (Spanish-validated version; English-non-validated version).
