# Supplementary material for: The Relationship Between Technology Use and Medication Access in Older Adults in Puerto Rico
Source: Int J Environ Res Public Health. 2025 Oct 7;22(10):1534. doi: 10.3390/ijerph22101534 (PMC12563701; doi:10.3390/ijerph22101534)
Supplement: Supplementary file 1 [file ijerph-22-01534-s001.zip › Final Questionnaire [English-non-validated version].pdf]

NON-VALIDATED TRANSLATION TO ENGLISH

University of Puerto Rico
Medical Sciences Campus
School of Pharmacy

Questionnaire to Measure the Relationship Among Technology Use and Medication Access in Older Adults in Puerto Rico.

Dear participant:

Thank you for your willingness to participate in this study. The purpose of this questionnaire is to gather information about your access to digitalized pharmaceutical services and your use of technology in daily life. Your participation is completely voluntary. You may choose to withdraw from the study at any time, without penalty or the need to explain your reasons. You may also refuse to answer any questions you do not wish to answer. If you have any questions while completing the questionnaire, please feel free to ask for assistance. Thank you for your time and collaboration.

Responses to the questionnaire are confidential; therefore, participants will not be individually identified. The information sheet, questionnaire, and results will be stored under lock and key, and only the researchers will have access to these documents. All materials will be destroyed and eliminated after three years.

If you have any questions or concerns related to the questionnaire, you may contact the Principal Investigator:

Dr. Jonathan Hernández Agosto
University of Puerto Rico
Medical Sciences Campus
Phone: 787-758-2525 ext. 5420
E-mail: jonathan.hernandez12@upr.edu

If you have any questions about your rights as a participant in the above-described study, you may contact:

Office for the Protection of Human Subjects in Research
University of Puerto Rico
Medical Sciences Campus
Phone: 787-758-2525 ext. 2510 o 2515
E-mail: opphi.rcm@upr.edu

Thank you for participating!

(This space has intentionally been left blank. Please pass to the next page to start the questionnaire.)

Control: ___: ___

General Instructions for the Questionnaire

Please read each question carefully before answering. There are no right or wrong answers; what matters is your personal experience. Your participation is entirely voluntary, and all your responses will be kept confidential. This questionnaire will take approximately 30 minutes to complete. You may withdraw from this study at any time, without providing a reason and without any penalty.

Section 1: Demographic Data:

In this section, we ask you to provide basic information about yourself. This will help us better understand your personal context. Please mark with an "X" the option that best describes you and provide specific answers to the open questions. If there are questions that do not apply to your situation, you may leave them blank.

1. Do you live alone or with others?

    - ☐ I live with my spouse
    - ☐ I live alone
    - ☐ I live with a partner
    - ☐ I live with family members
    - ☐ I live in a care residence (place that provides anything from basic assistance with hygiene and nutrition to specialized care, depending on the patient's needs)
    - ☐ Other: ___________________(specify)

2. Do you have the help of a caregiver or someone who regularly supports you with managing your health and the use/management of your medications?

    - ☐ Yes, only for health
    - ☐ Yes, only for medication management
    - ☐ Yes, for both
    - ☐ No

(This space was intentionally left blank. Please pass on to the next page to continue with the questionnaire)

3. How often do you visit a healthcare provider (e.g., doctor, nurse, pharmacist, etc.)?

    - ☐ Weekly
    - ☐ Monthly
    - ☐ Every 2 to 6 months
    - ☐ Annually
    - ☐ Only in emergencies

4. How often does a healthcare provider (e.g., doctor, nurse, pharmacist, etc.) visit your home?

    - ☐ Weekly
    - ☐ Monthly
    - ☐ Every 2 to 6 months
    - ☐ Annually
    - ☐ Only in emergencies

5. What is your municipality (town) of residence?

    Municipality (town): ___________________

6. What is your current employment status?

    - ☐ Employed (underline the one that applies: full time, part-time, business owner)
    - ☐ Retired
    - ☐ Disabled
    - ☐ Other: __________________ (specify)

(This space was intentionally left blank. Please pass on to the next page to continue with the questionnaire)

7. What is the highest level of education you have completed?

- ☐ Primary school
- ☐ Middle school
- ☐ High school
- ☐ Certification
- ☐ Associate degree
- ☐ Bachelor's degree
- ☐ Graduate degree
- ☐ No schooling

8. What is your income type?

- ☐ Individual
- ☐ Couple
- ☐ Other: ____________________(specify)

9. Based on your previous answer, what is your approximate monthly or annual income? [Indicate only one]

Monthly Income: __________________ Annual income: ____________________

10. With which gender do you identify?

- ☐ Male
- ☐ Female
- ☐ Non-binary (does not identify as male or female)
- ☐ Other: (specify)
- ☐ Prefer not to answer

11. What is your age?

Age:

Section 2: Technology Use

This section aims to learn more about your use of technology devices and access to the internet. Please respond based on your daily experience. If you do not frequently use technology, mark the appropriate option. Please mark with an “X” the option that best describes you. If there are questions that do not apply to your situation, you may leave them blank.

1. How would you describe your general ability to use technology?

   - ☐ Very skilled
   - ☐ Skilled
   - ☐ Slightly skilled
   - ☐ Not skilled

2. Do you have internet service access (“internet”, mobile data, Wi-Fi-) at home?

   - ☐ Yes
   - ☐ No

3. How often do you use the internet (“internet”, mobile data, Wi-Fi)?

   - ☐ Never
   - ☐ Once a month
   - ☐ Once a week
   - ☐ More than once a week
   - ☐ Once a day
   - ☐ More than once a day

4. What device do you use to access the internet? Select all that apply:

   - ☐ Mobile phone
   - ☐ Computer
   - ☐ Tablet
   - ☐ None

5. How often do you use technology devices for purposes NOT related to medication management (such as social media, email, banking, etc.)?

- ☐ Never
- ☐ Once a month
- ☐ Once a week
- ☐ More than once a week
- ☐ Once a day
- ☐ More than once a day

6. Have you used applications on a mobile device (e.g., WhatsApp, Facebook, Google Search)

- ☐ Yes
- ☐ No

(This space was intentionally left blank. Please pass on to the next page to continue with the questionnaire)

Section 3: Medication Access

In this final section, we want to know how the digitalization of pharmaceutical care has affected your ability to access medications and adhere to treatment. Please respond based on your personal experience with access to pharmacy services and your treatment adherence. Mark with an "X" the option that best describes you.

1. Do you use technology devices (such as a mobile phone, computer, or tablet) to receive pharmaceutical services?
   - ☐ Yes
   - ☐ No
2. How often do you receive notifications or automatic reminders from your pharmacy (e.g., text messages, emails, phone calls, etc.) related to the medications you use?
   - ☐ Never
   - ☐ Once a month
   - ☐ Once a week
   - ☐ More than once a week
   - ☐ Once a day
   - ☐ More than once a day
3. Do you use technology platforms (e.g., websites, mobile apps) to receive pharmaceutical services?
   - ☐ Yes
   - ☐ No
   - ☐ To my knowledge, my pharmacy does not use those technology tools
4. Have you ever been unable to request or pick up a medication because you could not use the technological tools (e.g., apps or websites) required by your pharmacy?
   - ☐ Yes
   - ☐ No
   - ☐ To my knowledge, my pharmacy does not use those technology tools
   - ☐ I do not remember

5. Do you think that the use of technology to obtain pharmaceutical services makes it easier for you to access your medication therapy?

    - ☐ Yes
    - ☐ No

6. What is your **preferred** option for obtaining **pharmaceutical services** (such as requesting medication refills, receiving medication counseling, scheduling vaccination appointments, etc.)?

    - ☐ Technology platforms (websites, mobile apps, etc)
    - ☐ Telephone call
    - ☐ Text message via mobile phone
    - ☐ For the physician to send my prescription directly to the pharmacy
    - ☐ Visiting the pharmacy in person
    - ☐ Other: ________________________ (specify)

You have completed the questionnaire. Please return the document to the research personnel or place it in the designated location.
