# Supplementary material for: The Relationship Between Technology Use and Medication Access in Older Adults in Puerto Rico
Source: Int J Environ Res Public Health. 2025 Oct 7;22(10):1534. doi: 10.3390/ijerph22101534 (PMC12563701; doi:10.3390/ijerph22101534)
Supplement: Supplementary file 1 [file ijerph-22-01534-s001.zip › Questionnaire_Final_v4 [Spanish-validated version].pdf]

Universidad de Puerto Rico
Recinto de Ciencias Médicas
Escuela de Farmacia

Cuestionario para medir la relación entre el uso de tecnología y el acceso a medicamentos en los adultos mayores de Puerto Rico

Estimado/a participante:

Gracias por su disposición para participar en este estudio. Este cuestionario tiene como objetivo recopilar información sobre su acceso a servicios farmacéuticos digitalizados y el uso de tecnología en su vida diaria. Su participación es completamente voluntaria. Usted puede decidir retirarse en cualquier momento, sin penalidad ni necesidad de explicar las razones. También puede negarse a contestar cualquier pregunta que no desee responder. Si tiene alguna pregunta mientras completa el cuestionario, no dude en pedir asistencia. Gracias por su tiempo y colaboración.

Las respuestas del cuestionario son confidenciales, por lo tanto, los participantes no serán identificados de manera individual. Tanto la hoja informativa, como el cuestionario, y sus resultados serán archivados bajo llave y solo tendrán acceso a los documentos los investigadores. Todo el material será destruido y eliminado luego de tres años.

De surgir cualquier pregunta o preocupación relacionada al cuestionario, puede contactar al Investigador Principal:

Dr. Jonathan Hernández Agosto
Universidad de Puerto Rico
Recinto de Ciencias Médicas
Teléfono: 787-758-2525 ext. 5420
E-mail: jonathan.hernandez12@upr.edu

De usted tener alguna pregunta sobre sus derechos como participante del estudio antes descrito, puede contactar a:

Oficina de Protección para Sujetos Humanos en Investigación
Universidad de Puerto Rico
Recinto de Ciencias Médicas
Teléfono: 787-758-2525 ext. 2510 o
2515 E-mail: opphi.rcm@upr.edu

¡Gracias por su participación!

(Este espacio se dejó intencionalmente en blanco. Favor pasar a la próxima página para comenzar el cuestionario.)

Instrucciones Generales del Cuestionario

Por favor, lea cuidadosamente cada pregunta antes de contestar. No hay respuestas correctas o incorrectas; lo importante es su experiencia personal. Su participación es completamente voluntaria y todas sus respuestas serán confidenciales. Este cuestionario tomará aproximadamente 30 minutos. Usted puede retirarse de este estudio en cualquier momento, sin necesidad de dar una razón y sin penalidad alguna.

Sección 1: Datos Demográficos Instrucciones:

En esta sección le pedimos que proporcione algunos datos básicos sobre usted. Esta información nos ayudará a entender mejor su contexto personal. Por favor, marque con una "X" la opción que mejor lo describa y provea la respuesta específica en las preguntas abiertas. Si hay preguntas que no aplican a su situación, puede dejarlas en blanco.

1. ¿Vive solo/a o acompañado/a?

   - ☐ Vivo con mi esposa/o
   - ☐ Vivo solo/a
   - ☐ Vivo con pareja
   - ☐ Vivo con familiares
   - ☐ Vivo en residencia de cuidado (lugar donde se provee desde asistencia básica con la higiene y alimentación hasta cuidados especializados dependiendo de las necesidades del paciente)
   - ☐ Otro: ___________________(especifique)

2. ¿Cuenta con la ayuda de algún cuidador o la asistencia de alguna persona que regularmente lo/la apoye con la gestión de su salud y el control/manejo de sus medicamentos?

   - ☐ Sí, solo para salud
   - ☐ Si, solo para control/manejo de medicamentos
   - ☐ Si, para ambos
   - ☐ No

(Este espacio se dejó intencionalmente en blanco. Favor pasar a la próxima página para continuar con el cuestionario.)

3. ¿Con qué frecuencia visita algún proveedor de salud (ej. médico, enfermero, farmacéutico, etc.)?

   - ☐ Semanalmente
   - ☐ Mensualmente
   - ☐ Cada 2 a 6 meses
   - ☐ Anualmente
   - ☐ Solo en emergencias

4. ¿Con qué frecuencia recibe visitas en su hogar de algún proveedor de salud (ej. médico, enfermero, farmacéutico, etc.)?

   - ☐ Semanalmente
   - ☐ Mensualmente
   - ☐ Cada 2 a 6 meses
   - ☐ Anualmente
   - ☐ Solo en emergencias

5. ¿Cuál es su municipio (pueblo) de residencia?

   Municipio (pueblo): ____________________

6. ¿Cuál es su estatus laboral actual?

   - ☐ Empleado (subraye el que aplique: tiempo completo, tiempo parcial o negocio propio)
   - ☐ Retirado
   - ☐ Incapacitado
   - ☐ Otro: ____________________(especifique)

(Este espacio se dejó intencionalmente en blanco. Favor pasar a la próxima página para continuar con el cuestionario.)

7. ¿Cuál es el nivel de educación más alto que ha completado?

- ☐ Escuela primaria
- ☐ Escuela intermedia
- ☐ Escuela superior
- ☐ Certificación
- ☐ Grado Asociado
- ☐ Bachillerato
- ☐ Postgrado
- ☐ Ninguna escolaridad

8. ¿Cuál es su tipo de ingreso?

- ☐ Individual
- ☐ Pareja
- ☐ Otro: ___________________(especifique)

9. Basado en su contestación anterior, ¿cuál es su ingreso mensual o anual aproximado? [Indique solo uno]

   Ingreso mensual: _______________ Ingreso anual: ____________________

10. ¿Con qué género se identifica usted?

- ☐ Masculino
- ☐ Femenino
- ☐ No binario (Que no se identifica como hombre o mujer)
- ☐ Otro: ___________________(especifique)
- ☐ Prefiero no responder

11. ¿Cuál es su edad?

   Edad: ____________

Sección 2: Uso de Tecnología

Esta sección tiene como objetivo conocer más sobre su uso de dispositivos tecnológicos y el acceso a internet. Por favor, responda con base en su experiencia diaria. Si no utiliza la tecnología con frecuencia, marque la opción correspondiente. Por favor, marque con una "X" <u>la opción que mejor lo describa</u>. Si hay preguntas que no aplican a su situación, puede dejarlas en blanco.

1. ¿Cómo describiría su habilidad al usar la tecnología en general?

   - ☐ Tengo mucha habilidad
   - ☐ Tengo habilidad
   - ☐ Tengo poca habilidad
   - ☐ No tengo habilidad

2. ¿Tiene acceso al servicio de internet (“internet”, datos móviles de su celular, wifi) desde su hogar?

   - ☐ Sí
   - ☐ No

3. ¿Con qué frecuencia usa el internet (“internet”, datos móviles de su celular, wifi)?

   - ☐ Nunca
   - ☐ Una vez al mes
   - ☐ Una vez a la semana
   - ☐ Más de una vez a la semana
   - ☐ Una vez al día
   - ☐ Más de una vez al día

4. ¿Qué dispositivo usa para acceder a internet? Marque todas las que apliquen:

   - ☐ Teléfono móvil
   - ☐ Computadora
   - ☐ Tableta
   - ☐ Ninguno

5. ¿Con qué frecuencia utiliza dispositivos tecnológicos para otros fines que **NO** se relacionan a la gestión de medicamentos (como redes sociales, correo electrónico, banca, etc.)?

   - ☐ Nunca
   - ☐ Una vez al mes
   - ☐ Una vez a la semana
   - ☐ Más de una vez a la semana
   - ☐ Una vez al día
   - ☐ Más de una vez al día

6. ¿H a usado aplicaciones en el dispositivo móvil (Ej. WhatsApp, Facebook, Buscador de Google)?

   - ☐ Sí
   - ☐ No

(Este espacio se dejó intencionalmente en blanco. Favor pasar a la próxima página para continuar con el cuestionario.)

Sección 3: Acceso a Medicamentos

En esta última sección, queremos saber cómo la digitalización del cuidado farmacéutico ha afectado su capacidad para acceder a sus medicamentos y la adherencia al tratamiento. Responda según su experiencia personal con el acceso a los servicios de farmacia y el seguimiento de su tratamiento. Por favor, marque con una "X" la opción que mejor lo describa.

1. ¿Utiliza dispositivos tecnológicos (como teléfono móvil, computadora o tableta) **para recibir servicios farmacéuticos**?

   - ☐ Sí
   - ☐ No

2. ¿Cuán frecuente recibe **de su farmacia** notificaciones o recordatorios automáticos (por ejemplo, mensajes de texto, correos electrónicos, llamadas telefónicas, etc.) relacionados a los medicamentos que utiliza?

   - ☐ Nunca
   - ☐ Una vez al mes
   - ☐ Una vez a la semana
   - ☐ Más de una vez a la semana
   - ☐ Una vez al día
   - ☐ Más de una vez al día

3. ¿Utiliza las plataformas tecnológicas (por ejemplo, sitios en internet, aplicaciones móviles) **para recibir servicios farmacéuticos**?

   - ☐ Sí
   - ☐ No
   - ☐ Hasta donde sé, mi farmacia no utiliza esas herramientas tecnológicas

4. ¿Alguna vez ha dejado de pedir o recoger un medicamento porque no pudo utilizar las herramientas tecnológicas (por ejemplo, aplicaciones o sitios web) que su farmacia requería?

   - ☐ Sí
   - ☐ No
   - ☐ Hasta donde sé, mi farmacia no utiliza esas herramientas tecnológicas
   - ☐ No recuerdo

5. ¿Piensa que el uso de la tecnología para obtener servicios farmacéuticos le facilita el acceso a su terapia de medicamentos?

   - ☐ Sí
   - ☐ No

6. ¿Cuál es su opción **favorita** para la obtención de **servicios farmacéuticos** (como pedir despacho de medicamentos, consejería en medicamentos, obtener citas para vacunas, etc.)?

   - ☐ Plataformas tecnológicas (sitios en internet, aplicaciones móviles, etc.)
   - ☐ Llamada telefónica
   - ☐ Mensaje de texto a través del móvil
   - ☐ Que el médico la envíe directamente a la farmacia
   - ☐ Visitando la farmacia en persona
   - ☐ Otro: _______________(especifique)

Ha concluido el cuestionario. Favor entregar el documento al investigador o colocarlo en el lugar provisto.
